# Recommended implementation of electrical resistance tomography for conductivity mapping of metallic nanowire networks using voltage excitation

**DOI:** 10.1038/s41598-021-92208-w

**Published:** 2021-06-23

**Authors:** Alessandro Cultrera, Gianluca Milano, Natascia De Leo, Carlo Ricciardi, Luca Boarino, Luca Callegaro

**Affiliations:** 1 Quantum Metrology and Nanotechnologies, INRIM - Istituto Nazionale di Ricerca Metrologica, 10135 Turin, Italy; 2 Advanced Materials Metrology and Life Sciences, INRIM - Istituto Nazionale di Ricerca Metrologica, 10135 Turin, Italy; 3 Department of Applied Science and Technology, Politecnico di Torino, 10129 Turin, Italy

**Keywords:** Energy science and technology, Materials science, Nanoscience and technology

## Abstract

The knowledge of the spatial distribution of the electrical conductivity of metallic nanowire networks (NWN) is important for tailoring the performance in applications. This work focuses on Electrical Resistance Tomography (ERT), a technique that maps the electrical conductivity of a sample from several resistance measurements performed on its border. We show that ERT can be successfully employed for NWN characterisation if a dedicated measurement protocol is employed. When applied to other materials, ERT measurements are typically performed with a constant current excitation; we show that, because of the peculiar microscopic structure and behaviour of metallic NWN, a constant voltage excitation protocols is preferable. This protocol maximises the signal to noise ratio in the resistance measurements—and thus the accuracy of ERT maps—while preventing the onset of sample alterations.

## Introduction

Electrical Resistance Tomography (ERT, and its counterpart in the ac regime, Electrical Impedance Tomography, EIT^1–3^) is a technique that determines a spatial map of the conductivity of an object by performing electrical resistance measurements between electrodes on its boundary. ERT was originally developed as a tool for physiology monitoring^4^ and earth science^5^, but later it has been successfully applied to the electrical characterisation of silicon wafer for electronics^6^, composite materials^7^, thin films^8^ and graphene^9^. ERT can provide accurate quantitative results in simple implementations with commercial instrumentation^8,9^; with dedicated hardware, it can reach a refresh speed in the ms range^10^. At the best of our knowledge, to date little evidence of electrical mapping of metallic nanowire networks (NWN) is available in literature^11^. On the contrary, a fast, non-destructive and traceable characterisation method like ERT would contribute to the establishment of novel NWN based technology. In fact, NWN have already attracted great interest as emerging transparent conductive materials, combining high transparency and flexibility with high electrical conductivity^12–17^. Consequently, these networks are being investigated for the development of transparent electrodes for photovoltaic applications^18,19^, transparent heaters^20^, flexible electronics^21^, but also for the realization of next-generation electronic devices exploiting the reconfigurability of NWN under electrical excitation^22–24^. These applications require the characterisation of the spatial homogeneity of the electrical properties of NWN at the mm scale or larger. In this sense ERT can become relevant for the development of reliable network-based electrodes and devices. Indeed, differently from in-line four-probe and van der Pauw measurements, ERT can provide information on local electrical properties. Typical ERT implementations, and also the one discussed in this work, allow for mapping the conductivity of samples at the mm scale. So ERT can complement, for example, conductive probe microscopy techniques^25^, that are usually exploited for conductivity mapping at the micro-nano scale, and other more recent mapping techniques such as the 1-probe mapping^11^. Moreover, since ERT is a non-scanning technique, the measurement time depends only on the number of contacts placed on the sample’s edge and not on the sample size, so in principle it can be scaled to map even larger NWN samples. At present, examples of ERT on other materials with samples of up to 20 cm lateral size have been successfully proposed^6,7^.

However, the implementation of ERT for the characterisation of this kind of nanomaterial requires particular attention to the measurement protocol since NWN can be altered by the same electrical stimulation required for their characterisation^23^. In fact the possible onset of local high electric fields during the measurements can lead to structural and morphological changes in nanowires (NW) and nanowire junctions (NW-junctions) due to electromigration phenomena, with consequent alteration of the electrical properties of the network^26–28^. In this work we show that (i) ERT can be successfully applied to measure conductivity maps of Ag NWN coatings; (ii) the typical implementation of ERT, which uses a constant current excitation, can very likely alter the samples even at low current levels, and (iii) an alternative ERT implementation, using a constant voltage excitation, is required when dealing with NWN. Moreover, while guidelines for the correct implementation of ERT in other fields are available^29,30^, a dedicated study in the field of nanostructured materials like NWN is still missing.

**Figure 1:**
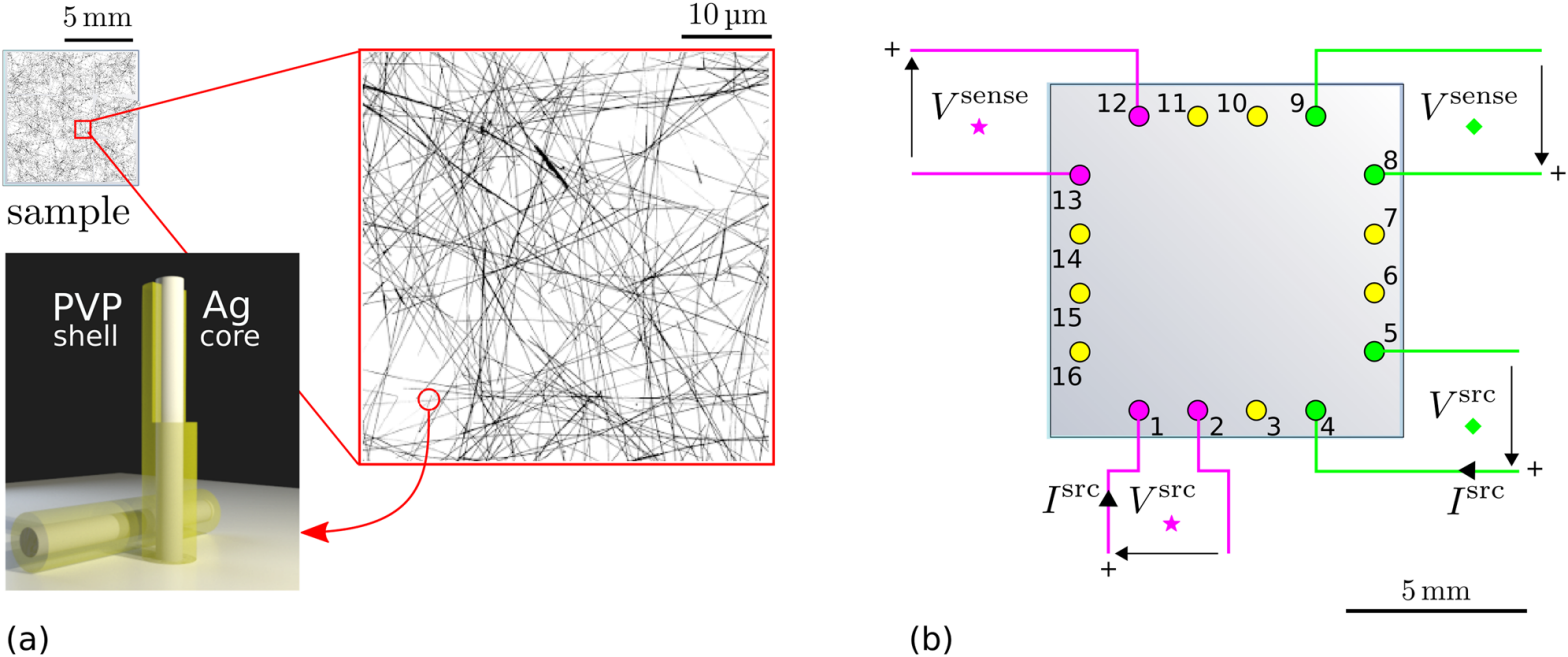
Nanowire network sample on quartz support: illustration of the $1\times 1\,\hbox {cm}^{2}$ sample, SEM micro-graph detail of one of the studied samples and a pictorial representation of PVP-Ag nanowires (**a**). Schematic representation of the placement of the contact array over the sample. Two examples of ERT four-terminal resistance measurements configurations are shown with the corresponding excitation and sensing quantities (**b**).

## Results

We used NWN made of silver nanowires grown by polyol synthesis process, drop-casted over quartz insulating substrates of $1\times 1\,\hbox {cm}^{2}$ in size (details in the experimental section “Methods”), resulting in a NWN with a highly interconnected morphology as shown in Fig. 1a. The measurements were performed within a few hours form the synthesis of the samples, to minimize possible degradation effects due to ambient exposure, which were previously observed to be negligible on this time scale^31^, and also are limited by the presence of the PVP shell^32^.

ERT conductivity maps are recovered performing a series of four-terminal transresistance measurements at the sample boundary, of the same type of those involved in the well-known van der Pauw^33^ method, and using a finite element model. Measurement and numerical methods are described in section “Methods” (see respectively the paragraphs ERT measurements and ERT modelling). The sample is contacted with an array of *n* probes at the sample boundary, see Fig. 1b.

In ERT, a set of *N* four-terminal resistance measurements

1 $$\begin{aligned} R_{i,j;k,l} = \frac{V^{\mathrm {sense}}_{k,l}}{I^{\mathrm {src}}_{i,j}} \end{aligned}$$

are performed, where $V^{\mathrm {sense}}_{k,l}$ is the voltage at contacts *k*, *l* and $I^{\mathrm {src}}_{i,j}$ the current flowing through contacts *i*, *j*.

In our application $n=16$, and we adopted the so-called *adjacent* measurement pattern^4^, which involves $N= n \times (n-3) = 208$ transresistance measurements. The typical ERT implementation^2^ adopts a *constant current* protocol ($\mathcal{I}_{{\mathrm{c}}}$ in the following): a current generator is set to source a constant current *I*, and is switched through all contact pairs, thus $I^{\mathrm{src}}_{i,j}=I$; the corresponding excitation voltage (often not measured) is $V^{\mathrm{src}}_{i,j}$. $V^{\mathrm{sense}}_{k,l}$ are measured by a switched voltmeter, or by several in parallel. We experimented also a *constant voltage* protocol ($\mathcal{V}_{{\mathrm{c}}}$ in the following): a constant excitation voltage $V^{\mathrm {src}}_{i,j} = V$ is employed, and the corresponding current $I^{\mathrm {src}}_{i,j}$ is measured by an additional ammeter in the setup. $V^{\mathrm {sense}}_{k,l}$ are measured like in the $\mathcal{I}_{{\mathrm{c}}}$ protocol. The choice of the excitation amplitude (*I* for the $\mathcal{I}_{{\mathrm{c}}}$ protocol, *V* for $\mathcal{V}_{{\mathrm{c}}}$) has effects both on the signal to noise ratio of the measurements, and on the occurrence of possible reconfiguration events in the NWN, as discussed in the following.

To make a consistent comparison between the two protocols $\mathcal{I}_{{\mathrm{c}}}$ and $\mathcal{V}_{{\mathrm{c}}}$, we can consider the total electrical energy *E* dissipated in the overall ERT measurement involving *N* transresistances:

2 $$\begin{aligned} E&= \sum _{m=1}^N V^{\mathrm {src}}_{i,j} I^{\mathrm {src}}_{i,j} T&\nonumber \\&= I T \sum _{m=1}^N V^{\mathrm {src}}_{i,j}&{\mathcal {I}}_{{\mathrm {c}}} \nonumber \\&= V T \sum _{m=1}^N I^{\mathrm {src}}_{i,j}&\mathcal{V}_{{\mathrm{c}}}, \end{aligned}$$

where *T* is the sample excitation time per individual transresistance measurement. *E* determines the signal to noise ratio of the overall ERT measurement.

For the ERT reconstruction process, the *N* transresistances given by Eq. (1) are casted in a vector ${\mathbf {R}} = [R_{i,j;k,l}]$ (see Fig. 3a). The conductivity map $\sigma$ is the solution of the variational problem

3 $$\begin{aligned} {\varvec\sigma } = \underset{ {\varvec\varsigma } }{{\text {arg}}\,{\text {min}}}\; \left( || {\mathbf {R}}_{\mathrm{calc}}( {\varvec\varsigma } ) - {\mathbf {R}})||^2 \right) , \end{aligned}$$

where ${\mathbf {R}}_{\mathrm{calc}}(\varsigma )$ is the vector of transresistances calculated using the guess conductivity distribution $\varsigma$ and $||\bullet ||$ is the Euclidean norm operator. Equation (3) is solved numerically.

We performed ERT measurements on several samples with both protocols $\mathcal{I}_{{\mathrm{c}}}$ and ${\mathcal {V}}_{{\mathrm {c}}}$ and increasing *E* levels, starting from $E\approx {3}\,\upmu \hbox {J}$; below this value transresistance measurements resulted too noisy to perform meaningful reconstructions.

**Figure 2:**
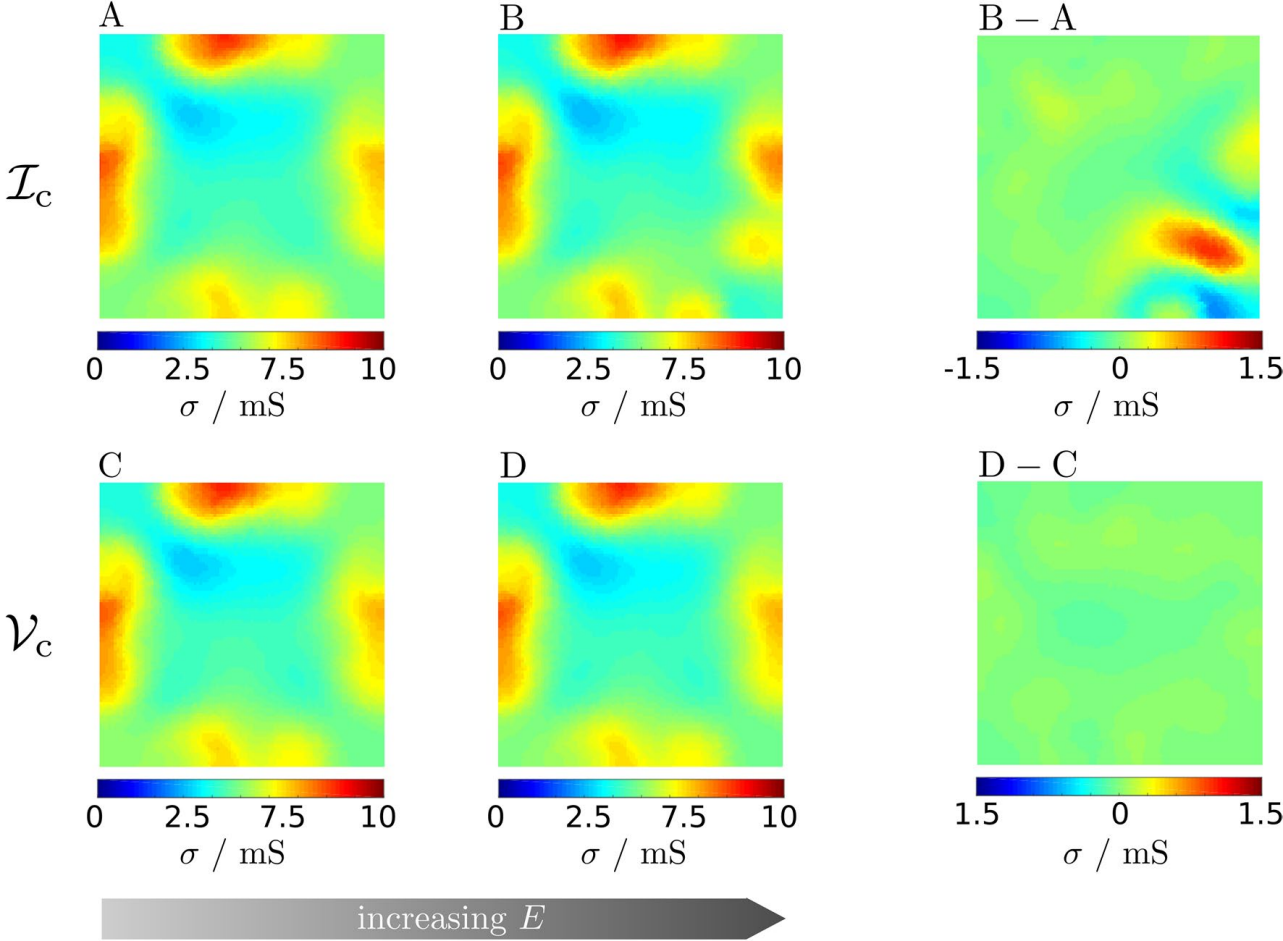
ERT electrical conductivity maps of sample S obtained with measurement protocols ${\mathcal {I}}_{{\mathrm {c}}}$ (constant current) and ${\mathcal {V}}_{{\mathrm {c}}}$ (constant voltage) at increasing excitation energy *E*. Maps ${\mathbf {A}}$ and ${\mathbf {C}}$ correspond to low sample excitation energy $E < {55}\,\upmu \hbox {J}$, while maps ${\mathbf {B}}$ and ${\mathbf {D}}$ correspond to higher sample excitation energy $E > {5.5}\,{\upmu \hbox {J}}$. Map difference ${\mathbf {B-A}}$ and ${\mathbf {D-C}}$ show the pixel-by-pixel absolute difference between the corresponding conductivity maps.

Figure 2 reports four ERT conductivity maps, ${\mathrm {A}}$, ${\mathrm {B}}$, ${\mathrm {C}}$ and ${\mathrm {D}}$, on a sample of areal mass density (AMD) ${105}\,\hbox {mg m}^{-2}$, named S in the following. The maps shown in the following reflect the peculiar NW distribution occurred on the considered sample, see Supplementary S3 for an example of a sample that shown a different conductivity distribution.

Measurement conditions are summarized in Table 1. Maps ${\mathrm {A}}$ and ${\mathrm {B}}$ were retrieved from measurements performed with protocol ${\mathcal {I}}_{{\mathrm {c}}}$; maps ${\mathrm {C}}$ and ${\mathrm {D}}$ with protocol ${\mathcal {V}}_{{\mathrm {c}}}$. The measurement parameters are given in Table 1.

Map ${\mathrm {A}}$ was obtained with protocol ${\mathcal {I}}_{{\mathrm {c}}}$ using a low excitation energy $E= {29.9}\,\upmu \hbox {J}$. The conductivity spans from 3.2 to ${8.3}\,\hbox {mS}$; the map average value, ${5.04}\,\hbox {mS}$, is consistent with other works^34^. Map ${\mathrm {C}}$ was obtained with protocol ${\mathcal {V}}_{{\mathrm {c}}}$, also with a low excitation energy $E={25.5}\,\upmu \hbox {J}$. Maps ${\mathrm {C}}$ and ${\mathrm {A}}$ are very similar, as can be quantified by the norm of the pixel-by-pixel difference $\delta _{\mathrm {XY}} = \frac{||{\mathrm {X-Y}}||}{||{\mathrm {Y}}||}$, where $||\bullet ||$ is the sum of the square of the conductivity (or conductivity difference) of each pixel (Frobenius norm), of only $\delta _{\mathrm {CA}} = {0.59}{\%}$ between maps ${\mathrm {C}}$ and ${\mathrm {A}}$. Protocols ${\mathcal {I}}_{{\mathrm {c}}}$ and ${\mathcal {V}}_{{\mathrm {c}}}$ gave equivalent results when performed at $E\approx {10}\,\upmu \hbox {J}$ on sample S.

Maps ${\mathrm {B}}$ and ${\mathrm {D}}$ correspond to measurements performed with a higher excitation energy, potentially of interest to improve the measurement signal to noise ratio, and hence the ERT map accuracy. Map ${\mathrm {B}}$ was obtained with protocol ${\mathcal {I}}_{{\mathrm {c}}}$ with $E = {7.8}\,\hbox {mJ}$. The difference with map ${\mathrm {A}}$ can be visually appreciated; Fig. 2 shows the map relative difference $\mathrm {B - A}$, which is up to of 20%, with $\delta _{{\mathrm {BA}}} = {3.50}{\%}$. Map ${\mathrm {D}}$ was obtained using protocol ${{\mathcal {V}}}_{{\mathrm {c}}}$ and $E = {7.1}\,\hbox {mJ}$, very close to that employed for map ${\mathrm {B}}$. By the way, the map relative difference $\mathrm {D - C}$ ranges within 1.3%, with a map difference as low as $\delta _{{\mathrm {DC}}} = {0.69}{\%}$. To summarize, maps ${\mathrm {A}}$, $\mathrm {C}$ and ${\mathrm {D}}$ are very similar; only map ${\mathrm {B}}$ shows a substantial deviation. Protocols ${\mathcal {I}}_{{\mathrm {c}}}$ and ${{\mathcal {V}}}_{{\mathrm {c}}}$ gave no more equivalent results when performed on S at excitation energy $E> {2.6}\,\hbox {mJ}$.

**Table 1:** ERT settings and relevant quantities corresponding to the sample S. For each map shown in Fig. 2 are reported: the protocol, the excitation quantities, the total energy dissipated during the measurement and the map difference with respect to the chosen reference.

ERT map	Protocol	Excitation		*E*	$\delta _{{\mathrm {X,Y}}} / \%$
A	${\mathcal {I}}_{{\mathrm {c}}}$	$I={10}\,\upmu \hbox {A}$	$\max (V^{\mathrm {src}})={15}\,\hbox {mV}$	${29.9}\,\upmu \hbox {J}$	–
B	${\mathcal {I}}_{{\mathrm {c}}}$	$I={400}\,\upmu \hbox {A}$	$\max (V^{\mathrm {src}})={331}\,\hbox {mV}$	${7.8}\,\hbox {mJ}$	$\delta _{{\mathrm {B,A}}} = 3.50$
C	${\mathcal {V}}_{{\mathrm {c}}}$	$V={5}\,\hbox {mV}$	$\max (I^{\mathrm {src}})={124}\,\upmu \hbox {A}$	${25.5}\,\upmu \hbox {J}$	$\delta _{{\mathrm {C,A}}} = 0.59$
D	${\mathcal {V}}_{{\mathrm {c}}}$	$V={100}\,\hbox {mV}$	$\max (I^{\mathrm {src}})={780}\,\upmu \hbox {A}$	${7.1}\,\hbox {mJ}$	$\delta _{{\mathrm {D,C}}} = 0.69$

**Figure 3:**
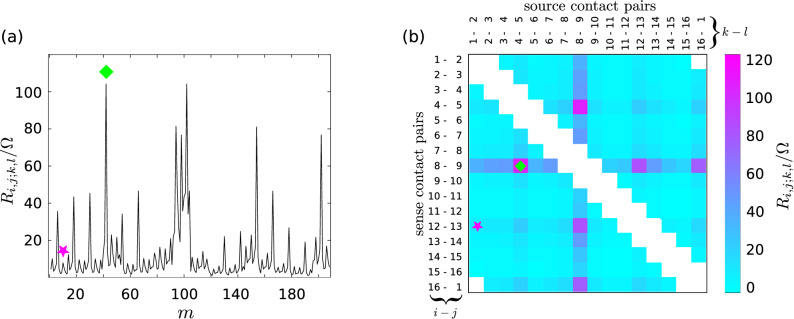
Representations of ERT data of the sample S. Plot of ${\mathbf {R}}$ relative to the map ${\mathrm {A}}$ in Fig. 2a. The same data represented as an impedance matrix ${\mathbf {Z}}$ (**b**). The diamond and star markers indicates the values of $R_{i,j;k,l}$ measured with the corresponding contact configurations shown in the scheme in Fig. 1b. Due to the measurements arrangement in the plot (**a**), the symmetry of the data is not evident as it happens looking at ${\mathbf {Z}}$ in (**b**).

A more direct analysis of the effect of the application of protocols ${\mathcal {I}}_{{\mathrm {c}}}$ or ${\mathcal {V}}_{{\mathrm {c}}}$ at different *E* levels can be made by considering the measured transresistances. Figure 3a shows a plot of the values $R_{i,j;k,l}$ corresponding to map ${\mathrm {A}}$ of Fig. 2; index $m = 1 \ldots N$ is the sequential index. The specific $R_{i,j;k,l}$ measured with the adjacent pattern correspond to contact configurations following the rules $j=i+1$ and $l=k+1$ (with the convention that $n+1 \equiv 1$), and can be recast in a matrix ${\mathbf {Z}}$ having as elements

4 $$\begin{aligned} Z_{ik} = R_{i,i+1;k,k+1} \qquad i,j = 1 \ldots n. \end{aligned}$$

${\mathbf {Z}}$ for map ${\mathrm {A}}$ is shown in Fig. 3b. The $N=208$ measurements leave some elements of the $16 \times 16$ matrix undefined; these correspond to two- or three-terminal measurement configurations which are not considered in ERT because they include the probe to sample contact resistances. In Fig. 3b a strong symmetry of ${\mathbf {Z}}$ respect to the diagonal can be visually appreciated.

The elements of ${\mathbf {Z}}$ symmetric respect to the diagonal, $Z_{pq} = R_{p,p+1;q,q+1}$ and $Z_{qp} = R_{q,q+1;p,p+1}$ correspond to configurations where source and sense terminals are swapped. The contacted sample can be considered a multiterminal electrical passive network, for which the reciprocity theorem^35^ $Z_{pq} \equiv Z_{qp}$ holds; hence, ${\mathbf {Z}}$ must be symmetric. Any deviation from symmetry is an indication of measurement errors, or alterations of the sample electrical properties caused by the measurement process. Let us quantify the overall asymmetry of a generic ${\mathbf {Z}}$. Since ${\mathbf {Z}}$ is square, the Toeplitz decomposition in a symmetric and skew-symmetric matrices applies:

5 $$\begin{aligned} {\mathbf {Z}} = \frac{1}{2} ({\mathbf {Z}} -{\mathbf {Z}}^\intercal ) + \frac{1}{2} ({\mathbf {Z}} + {\mathbf {Z}}^\intercal ), \end{aligned}$$

A symmetry index $\nu$ can be introduced as

6 $$\begin{aligned} \nu = \frac{||{\mathbf {Z}} - {\mathbf {Z}}^\intercal ||}{||{\mathbf {Z}} + {\mathbf {Z}}^\intercal ||}, \end{aligned}$$

where $||\bullet ||$ indicates a Frobenius norm, evaluated on the available ${\mathbf {Z}}$ entries. $\nu$ is dimensionless and $\ge 0$; if ${\mathbf {Z}}$ is symmetric, then $\nu$ $=0$, and it increases with increasing asymmetry.

**Figure 4:**
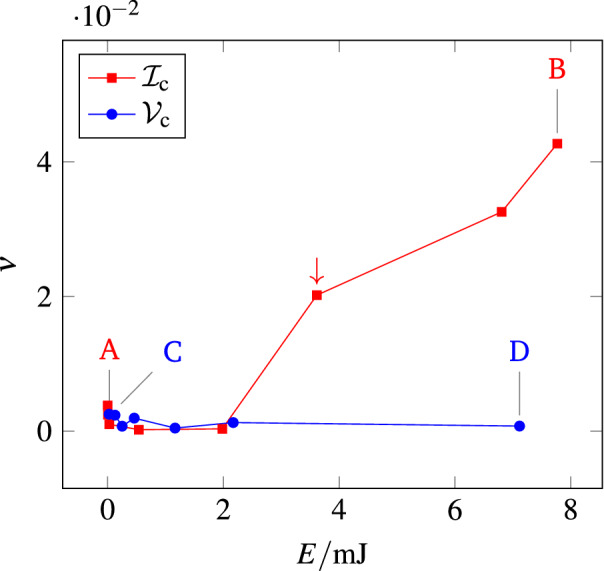
The symmetry index $\nu$ of sample S calculated from ERT measurements for increasing *E* performed at ${\mathcal {I}}_{{\mathrm {c}}}$  and ${\mathcal {V}}_{{\mathrm {c}}}$  . Letters and arrow label the values of $\nu$ corresponding to the maps shown and discussed in the text.

Figure 4 plots the asymmetry index $\nu$ as a function of *E*, ranging between ${18}\,\upmu \hbox {J}$ and ${7.8}\,\hbox {mJ}$, for the two measurement protocols ${\mathcal {I}}_{{\mathrm {c}}}$ and ${\mathcal {V}}_{{\mathrm {c}}}$. The values of $\nu$ corresponding to maps ${\mathrm {A}},{\mathrm {B}}, \mathrm {C}, {\mathrm {D}}$ of Fig. 2 are marked with letters.

## Discussion

The $\nu$ values corresponding to the ${\mathcal {V}}_{{\mathrm {c}}}$ protocol stay close to zero (hence, ${\mathbf {Z}}$ is symmetric) in the whole energy range investigated. Considering the ${\mathcal {I}}_{{\mathrm {c}}}$ protocol instead, $\nu$ is close to zero up to a critical energy $E_{{\mathrm {crit}}}\approx {0.8}\,\hbox {mJ}$, after which it ramps up, indicating an increasing asymmetry of ${\mathbf {Z}}$.

Figure 4 is consistent with Fig. 2: while maps ${\mathrm {A}}$, $\mathrm {C}$ and ${\mathrm {D}}$, all three corresponding to $\nu$ $\approx 0$, are very similar one another, the map ${\mathrm {B}}$, which shows differences, correspond to $\nu$ $>0$. The asymmetric ${\mathbf {Z}}$ of map ${\mathrm {B}}$ indicates an alteration occurring *in the course* of the measurement; note that if the sample became altered *during* the ERT measurement, then map ${\mathrm {B}}$ does not even represent a meaningful reconstruction of the conductivity distribution over the sample. To better show how changes in the actual ERT measurements (${\mathbf {Z}}$ of Fig. 3) produce changes in the conductivity maps shown in Fig. 2, in Supplementary S2 we reported the matrices ${\mathbf {Z}}$ corresponding to the four maps ${\mathrm {A}}$, ${\mathrm {B}}$, $\mathrm {C}$, ${\mathrm {D}}$, the differences $\mathrm {B-A}$ and $\mathrm {D-C}$.

It is noteworthy that $I^{\mathrm {src}}$ is not a good parameter to express an alteration threshold: considering the values of $I^{\mathrm {src}}$ achieved at maximum *E* (see Table 1), the maximum $I^{\mathrm {src}}$ applied in the ${\mathcal {V}}_{{\mathrm {c}}}$ protocol is ${780}\,\upmu \hbox {A}$ (case ${\mathrm {D}}$), while for the ${\mathcal {I}}_{{\mathrm {c}}}$ protocol it is of only ${400}\,\upmu \hbox {A}$ (case ${\mathrm {B}}$).

When performing measurements with ${\mathcal {I}}_{{\mathrm {c}}}$ protocol, the value of $E_{{\mathrm {crit}}}$ has an intrinsic variability, likely dependent on the NWN individual morphology. Measurements performed on other samples found NWN of AMD $\approx {85}\,\hbox {mg m}^{-2}$ with $E_{{\mathrm {crit}}}$ spanning between ${500}\,\upmu \hbox {J}$ and ${2}\,\hbox {mJ}$; for samples with AMD $\approx {115}\,\hbox {mg m}^{-2}$, between ${4.5}\,\hbox {mJ}$ and ${14.5}\,\hbox {mJ}$, a result consistent with a denser network. To this large variability corresponded different ERT maps, corroborating the fact that the individual NW distribution is relevant for the value of $E_{{\mathrm {crit}}}$. The measurements performed with ${\mathcal {V}}_{{\mathrm {c}}}$ protocol on these samples, instead, showed $\nu$ $\approx 0$ within the whole corresponding energy span. Additionally we observed that also measurements on samples of AMD $\approx {150}\,\hbox {mg m}^{-2}$, even though less systematically, showed the same criticality concerning the ${\mathcal {I}}_{{\mathrm {c}}}$ protocol, indicating that the ${\mathcal {V}}_{{\mathrm {c}}}$ protocol is recommended also for the characterisation of higher AMD NWN.

The observed sample alterations can be related to a reconfiguration of the NWN associated with electromigration phenomena occurring at the nanowire junctions. In fact, it has been extensively reported^23,27,28,36^ that applied voltage above a threshold around ${100}\,\hbox {mV}$ leads to dissolution of Ag atoms to form Ag+ ions that subsequently migrate under the action of the applied electric field in correspondence of NW junctions. The electromigration is responsible for the reconfiguration (alteration of the structure and morphology) of NW junctions, with consequent change in the NW junction resistance^24^, and in the end to the change in the NWN conductivity distribution probed by ERT. Low-resistance junctions can sustain currents of tens of $\upmu \hbox {A}$ each^23,28^, which is also consistent with our data for ${\mathcal {V}}_{{\mathrm {c}}}$ (see maximum excitation currents for ${\mathcal {V}}_{{\mathrm {c}}}$ in Table 1).

When performing an ERT measurement with the ${\mathcal {I}}_{{\mathrm {c}}}$ protocol, $\max (V^{\mathrm {src}})$ is not under control; in fact the onset of the alterations (marker  in Fig. 4), occurring at $E= {3.6}\,\hbox {mJ}$, corresponds to a maximum applied voltage, for the whole set of *N* electrode configurations, $\max (V^{\mathrm {src}}) = {240}\,\hbox {mV}$. The protocol ${\mathcal {V}}_{{\mathrm {c}}}$ allows to choose a $V^{\mathrm {src}}$ below the damage threshold and at the same time to maximize *E*, consequently maximizing the signal to noise ratio of the measured transresistance set ${\mathbf {R}}$ and hence the quality of the ERT reconstruction.

## Methods

### Metallic nanowire network synthesis

The investigated NWN were synthesized starting from Ag NWs (Sigma-Aldrich, prod. no. 739448) of length ${20}\,\upmu \hbox {m}$ to ${50}\,\upmu \hbox {m}$, diameter $115\pm (15)\hbox {nm}$, coated with 1–2 nm of PVP^24^ and concentration 0.5 wt.% in isopropyl alcohol (IPA). By the dilution of the original suspension by additional IPA we obtained NWN of AMD in the range between ${70}\,\hbox {mgm}^{-2}$ and ${120}\,\hbox {mgm}^{-2}$ (calculated). NWs were dispersed on a square quartz substrate of size $1 \times 1\,\hbox {cm}^{2}$. A detailed structural and chemical characterisation of these nanostructures is reported in our previous work^23^.

### ERT measurements

The measurement setup used in the present work allows to contact $1\times 1\,\hbox {cm}^{2}$ samples on rigid insulating substrates without any lithographic step. The sample is contacted by 16 gold-coated spring-mounted needle probes (FIXTEST GmbH, model 820.05.01.015) on a circular spot of $\approx {40}\,\upmu \hbox {m}$ in diameter. The sample is placed in a custom contact fixture and kept in place with an engraved plastic block. A rail-mounted mechanism is actuated to push the sample against the probe-array and achieve electric contact^9^. The probes are arranged along the sample edge (at ${500}\,\upmu \hbox {m}$ distance from it) as shown in Fig. 1b. The inter-probe spacing is 2 mm. Calibrated commercial instrumentation is used to perform the electrical measurements. A Keithley 2602B source-meter is used to generate $I^{\mathrm {src}}_{i,j}$ or $V^{\mathrm {src}}_{i,j}$ (and measure the other parameter); an Agilent 34461A voltmeter measures $V^{\mathrm {sense}}_{k,l}$. An Agilent 34980A mainframe, provided with a reed relay module 34933A is used to route the source-meter and voltmeter connections to the various contact configurations *i*, *j*; *k*, *l* according to the chosen measurement excitation pattern. To minimize electrical stresses on the sample, in particular when the ${\mathcal {I}}_{{\mathrm {c}}}$ protocol is chosen, each individual configuration *i*, *j*; *k*, *l* is reached by setting $I^{\mathrm {src}}$ (or $V^{\mathrm {src}}$) to zero, by operating a make-before-break switching, then setting back $I^{\mathrm {src}}$ (or $V^{\mathrm {src}}$) to the desired value *I* (or *V*) to perform the measurement. (See Supplementary S1 for a detailed ERT measurement setup schematic and thorough description of the adjacent measurement pattern).

For each measurement configuration *i*, *j*; *k*, *l*, eight measurements were performed and averaged, by reversing each time the excitation and sensing polarity in order to minimize instrument offset and noise. The total time (excitation and instruments switching) of a complete cycle of 208 ERT measurements was 275 s. The measurement setup is hosted in a controlled temperature laboratory at $(22\pm 1)^{\circ }\hbox {C}$, with a relative humidity of $(51\pm 5){\%}$.

### ERT modelling

ERT map reconstruction considers a continuous medium having a spatial conductivity function ${{\varvec{\sigma }}}$; the typical resolution of the reconstruction is of the order of the electrode spacing. The NWN considered are discontinuous at the scale of the wire interconnections ($<{10}\,\upmu \hbox {m}$) but can be safely considered continuous at the scale of the map resolution (around the mm); more details are given in Supplementary S4 using arguments from the related literature^37,38^.

ERT is an ill-posed inverse problem, sensitive to experimental noise in the input data. For this reason, the problem 3 is regularised by an additional term, resulting in

7 $$\begin{aligned} {\varvec\sigma } = \underset{ {\varvec\varsigma }}{{\text {arg}}\,{\text {min}}}\;\left( || {\mathbf {R}}_{{\mathrm {calc}}}( {\varvec\varsigma } ) - {\mathbf {R}})||^2 + \lambda || \mathbf {L}(\varsigma )||^2\right) , \end{aligned}$$

where $\lambda$ is the *regularization parameter*, a scalar used to set the amount of regularization.

Equation (7) must be solved numerically. We employed the open source function library EIDORS 3.7.1, which runs on MATLAB^39,40^. A two-dimensional, finite element model (FEM) of the sample was generated, with the contacts modeled as points corresponding to mesh nodes. $\mathbf {L}(\varsigma )$ is given by the EIDORS’ regularisation matrix Laplace_prior, which implements an approximation of the second derivative of $\varsigma$. To select $\lambda$ with an objective criterion we used the L-curve method^41,42^. The FEM model, having ${\mathbf {R}}$ as input data, is solved for $\mathbf {\varsigma }$ with an absolute-type Gauss-Newton an absolute-type Gauss–Newton inverse solver. The conductivity maps shown in this work were obtained interpolating the FEM solution $\sigma$ to a square grid of $100 \times 100$ pixels. The number of pixels was chosen to be not less than the FEM elements in the mesh (9554) in order to avoid interpolation artifacts.

## Conclusions

Electrical Resistance Tomography is usually performed using a constant current ${\mathcal {I}}_{{\mathrm {c}}}$ protocol. This protocol is not suitable for characterisation of NWN, since it can induce changes in the sample, due to polarization, even at low excitation levels. We showed that a constant voltage excitation protocol ${\mathcal {V}}_{{\mathrm {c}}}$ can be successfully employed to perform ERT maps on NWN without observable sample alteration. The protocol allows to maximize the sample excitation energy *E*, and hence the signal to noise ratio, for maximum ERT performance.

A quantification of the sample alteration during measurement can be obtained by considering an asymmetry index $\nu$ of the impedance matrix ${\mathbf {Z}}$.

The measurement procedure and criteria identified can be employed also in the charactersation of other types of NWN and other samples^43^ and in the ERT of other samples sensitive to damages due to excessive electrical excitation.

## Supplementary Information

Supplementary Information.
