# Supplementary material for: Recommended implementation of electrical resistance tomography for conductivity mapping of metallic nanowire networks using voltage excitation
Source: Sci Rep. 2021 Jun 23;11:13167. doi: 10.1038/s41598-021-92208-w (PMC8222310; doi:10.1038/s41598-021-92208-w)
Supplement: Supplementary file 1 — Supplementary Information. [file 41598_2021_92208_MOESM1_ESM.pdf]

Supplementary Information — Recommended implementation of electrical resistance tomography for conductivity mapping of metallic nanowire networks using voltage excitation

Alessandro Cultrera[1,*], Gianluca Milano[2,3], Natascia De Leo[2], Carlo Ricciardi[3], Luca Boarino[2], and Luca Callegaro[1]

[1]*INRIM — Istituto Nazionale di Ricerca Metrologica, Quantum Metrology and Nanotechnologies, Torino, 10135, Italy*
[2]*INRIM — Istituto Nazionale di Ricerca Metrologica, Advanced Materials Metrology and Life Sciences, Torino, 10135, Italy*
[3]*Politecnico di Torino, Department of Applied Science and Technology, Torino, 10129, Italy.*
[*]*a.cultrera@inrim.it*

Supplementary information S1: ERT measurement setup schematic and measurement pattern.

The number of ERT measurement configurations on a system with n electrodes depends on the implemented measurement protocol. Following the adjacent pattern, our system with $n = 16$ provides $N = n(n-3)$ four-terminal measurements, so the present ERT measurements involve $N = 208$ four-terminal resistance measurement configurations. The N measurements are stored in the transresistance vector $\mathbf{R}$.

Measurements performed according to the adjacent pattern involves only pairs of adjacent contacts, and each contact pair is used in sequence to energize the sample (applying I^{src} or V^{src}) while all the other pairs are used for sensing (measuring V^{sense}). Given four contacts $i, j; k, l$, the sample is

energized at contacts i, j and probed at contacts k, l, corresponding to the measurements $R_{i,j;k,l}$.

In Fig. S1-a is shown a schematic diagram of the switching device which connects the instrumentation to the sample holder. The white (coloured) dots represent open (closed) switch relays used to set the N contact configurations. In the example the configuration $(2, 3; 16, 1)$ is represented.

In Fig. S1-b the same contact configuration where contact (2,3) are used for the excitation is better explained. In particular are represented measurement configurations from $m = 14$, corresponding to configuration $(2, 3; 4, 5)$, to $m = 27$, corresponding to configuration $(2, 3; 16, 1)$, where contacts $(2, 3)$ are used as source pair. The dashed blue arrow represents the switching sequence for the sensing pairs. As it can be seen, in the case of the adjacent protocol with 16 contacts for each source pair i, j there are 13 sensing configurations k, l to be measured. Once the sample transresistance is measured in all the 13 available measurement configurations, the source pair is switched to the next pair $i+1, j+1$ and the sensing configuration are shifted accordingly.

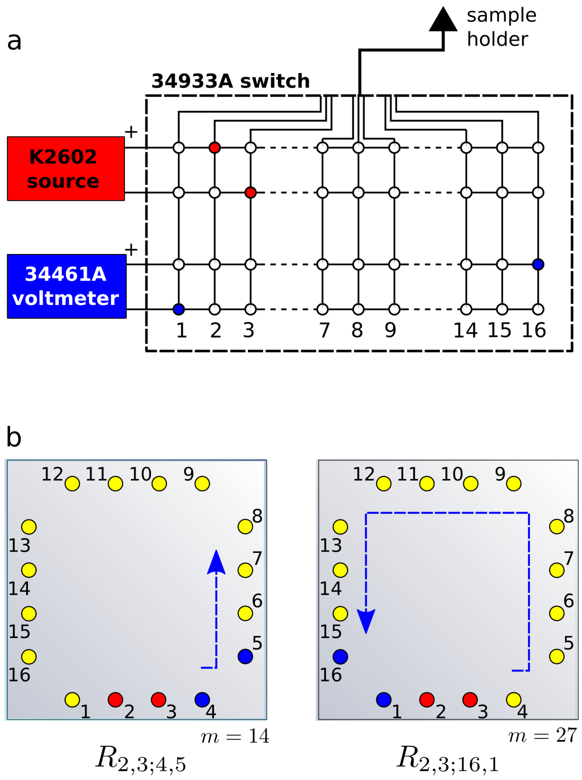

Figure S1: Scheme of the measurement setup (a). For sake of clarity only a part or the 16 contact connections are shown. The relays are set accordingly to the configuration $(2, 3; 16, 1)$. Contacts $(2, 3)$ in red colour are used as source pair. Sensing pairs are in blue. Working principle of the ERT adjacent measurement protocol with 16 contacts. The dashed arrow represents the verse in which the sensing contact pairs are sequentially probed during the measurements. Measurement configurations $(2, 3; 4, 5)$ and $(2, 3; 16, 1)$ are shown (b). For both frames here shown, the measurement index m and the corresponding transresistance are indicated.

Supplementary information S2: Z matrix corresponding to the maps A, B, C and D

To better show how changes in the actual ERT measurement lead to changes in the conductivity maps, we reported in Fig. S2 the matrices **Z** corresponding to the four maps A, B, C and D and their differences B − A and D − C

(Fig. 2 of the main text). The matrices are of the same type of the one shown and described in Fig. 3-b of the main text. The matrices in Fig. S2-A and -B correspond to ERT data obtained with the protocol $\mathcal{I}_{\rm c}$, while the matrices in Fig. S2-C and -D correspond to ERT data obtained with the protocol $\mathcal{V}_{\rm c}$. It can be noted that the difference in ERT data $\rm B - A$ is substantial, while the difference $\rm D - C$ is very small (always less that $0.8\,\Omega$, which is compatible with the measurement error at very low energy). Consistently, to a larger difference in ERT data corresponds a larger change in the conductivity maps.

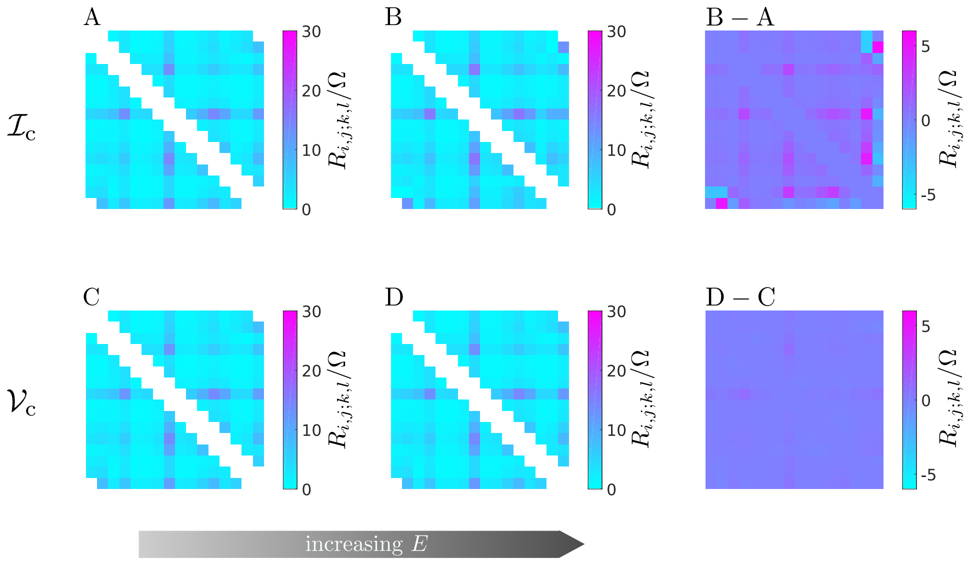

Figure S2: ERT data of sample S obtained with measurement protocols $\mathcal{I}_{\rm c}$ (constant current) and $\mathcal{V}_{\rm c}$ (constant voltage) at increasing excitation energy E. Matrices in A and C correspond to low sample excitation energy $E < 55\,\mu\rm J$, while matrices in B and D correspond to higher sample excitation energy $E > 5.5\,\rm mJ$. Matrices $\rm B - A$ and $\rm D - C$ show the difference between the corresponding ERT data sets.

Supplementary information S3: Example of a different NW distribution on another studied sample

The ERT maps A, B, C and D, presented in Fig. 2 of the main text, show the particular NW distribution of thats sample. Other samples, of the same

nominal AMD and size, once the NWN dispersion was deposited on the quartz substrate, shown different NW distributions. In Fig. S3 the ERT map of a sample is shown, where it can be seen that the NW distribution is more concentrated in the center.

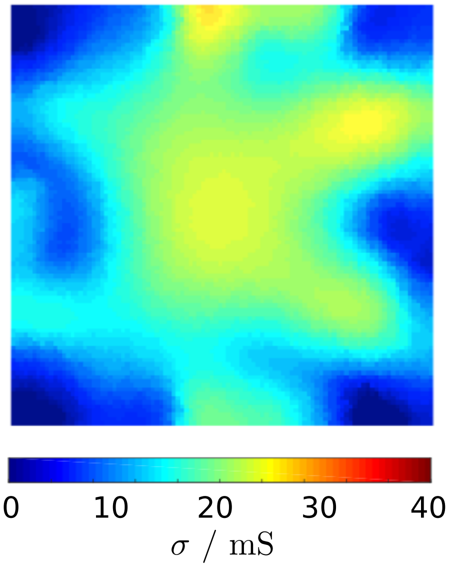

Figure S3: ERT map of another NWN sample synthesized following the same process presented in the main text. The size of the sample is $1 \times 1\ \mathrm{cm}^2$.

Supplementary information S4:
Validity of the ERT approach for metallic nanowire networks

Electrical resistance tomography is framed in the context of continuous media, described by a spatial conductivity function σ (see equation 7 in the main text). Since NWN have a microscopic structure, the validity of the ERT approach should be considered.

The electrical behaviour of NWN having properties very similar to those considered in this paper have been extensively modelled in [37], which analyses their behaviour in terms of a normalized density D, which takes the value $D_\mathrm{c} = 5.63$ at the percolation threshold. Fig. 1b of [37] shows that when $D > 2D_\mathrm{c}$ the continuous approximation is valid over a scale of just

a few nanowire lengths. This is consistent with resistance network models; for example, according to [38, Sec. VIII, p. 1003] the resistance of an infinite discrete network of equal resistors matches that of a continuous uniform sheet to a few part in a thousand when the resistance is measured just 3 or 4 lattice spacings apart.

All samples investigated in our work have $D > 25$ if considering an average nanowire length $L = 35\,\mu\mathrm{m}$ (as from the material specifications provided by the manufacturer). The ERT measurements presented are based on measurements performed over the whole sample size ($10\,\mathrm{mm}$) and for electrode spacings of $2\,\mathrm{mm}$. Since the spatial resolution of the reconstruction is of the order of the electrode spacing, the modelling of a NWN as a continuous medium for ERT reconstruction is acceptable.

More generally, the electrical behaviour of NWN as a quasi-continuous medium is widely exploited in applications, such as large-area transparent conductive coating for photovoltaic [17, 19], heaters [17, 20] and touch sensors [16 18, 21].

Bibliography

See main text.
